# Scalp Hair Regrowth in Hormone-Treated Transgender Woman

**DOI:** 10.1089/trgh.2016.0022

**Published:** 2016-10-01

**Authors:** Mary O. Stevenson, Naomi Wixon, Joshua D. Safer

**Affiliations:** ^1^ Department of Medicine, Boston Medical Center, Boston, Massachusetts.; ^2^ Worcester Polytechnic Institute, Worcester, Massachusetts.; ^3^ Center for Transgender Medicine and Surgery, Boston Medical Center, Boston University School of Medicine, Boston, Massachusetts.

**Keywords:** clinical care, hormone therapy, male pattern baldness, transgender, trans woman

## Abstract

Evidence of androgenetic alopecia, or male pattern baldness, can be distressing for transgender women. Here we present the case of a transgender woman with scalp hair regrowth after ∼6 months on oral estradiol and spironolactone therapy achieving testosterone levels within normal female range.

## Introduction

Androgenetic alopecia, also known as male pattern baldness, is a process by which hair loss from the scalp occurs in a progressive, predictable pattern. Recent research has proposed that it is a result of the presence of sufficient androgen, specifically dihydrotestosterone (DHT), in a person with genetic predisposition.^1^ Without therapeutic intervention, hair loss will become more severe over time.^2^ There are currently only two FDA-approved treatments for androgenetic alopecia and both are more effective in arresting hair loss than in reversing it.^1^ The first is minoxidil, a topical vasodilatory agent that was initially approved for use as an antihypertensive.^3^ The second is finasteride, an oral inhibitor of type 2 5α-reductase enzyme, thereby halting the conversion of testosterone to DHT^4^ and reducing DHT concentrations in both the serum and scalp.^5^ With both of these therapies, the cessation of treatment leads to a reinitiation of the balding process, demonstrating that their effects are not permanent.^2^

Although scalp hair loss can be unwanted and bothersome for some men, it can be particularly distressing for transgender women as it may serve as a physical sign of an undesired male phenotype. Here we present the case of a transgender woman with scalp hair regrowth while on hormone therapy with oral estradiol and spironolactone.

## Case Presentation

A 33-year-old transgender female presented to an outpatient clinic with hair thinning in addition to hair line regression at the crown and bilateral temporal regions of the scalp. She wished to reverse these changes to whatever extent possible.

**FIG. 1.**
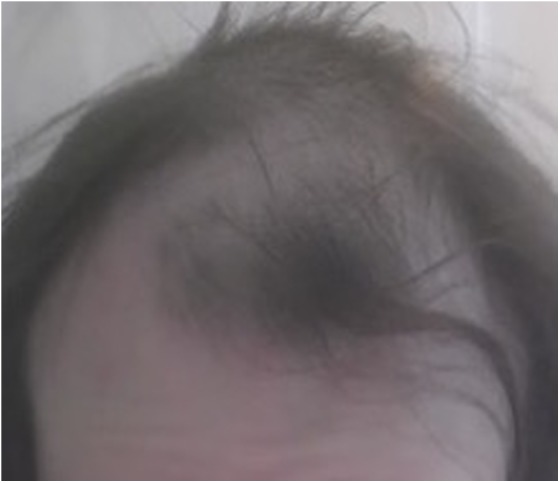
Scalp at baseline, frontal view. Serum testosterone level was 455 ng/dL.

**FIG. 2.**
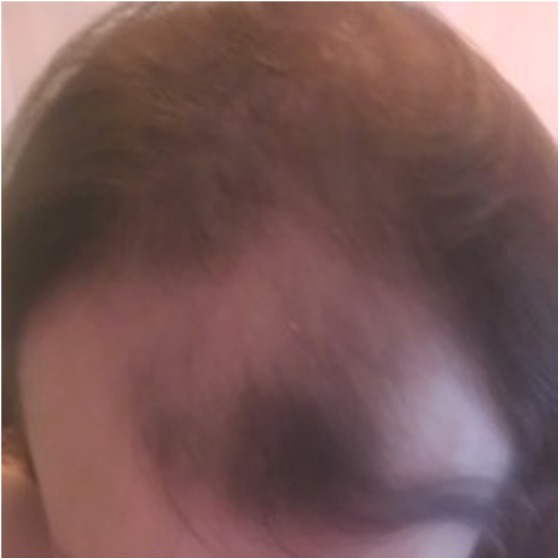
Scalp at baseline, top view.

The patient had a history of asthma. Medications were albuterol inhaler as needed. She had no known allergies. On examination, her vital signs were within normal limits. She was well appearing and in no acute distress. Her thyroid, pulmonary, cardiac, gastrointestinal, and neurological examinations were all within normal limits. She exhibited evidence of male pattern baldness with deep symmetrical recession of scalp hair at the temples as well as the frontal hairline (Figs. 1 and 2).

Baseline testosterone level was within normal male range (300–1000 ng/dL) at 455 ng/dL. After the patient completed sperm banking, she was started on oral estradiol and spironolactone therapy. Over the course of ∼1 year, hormone therapy was titrated until the patient's testosterone levels were at goal. On a stable dose of estradiol 5 mg daily and spironolactone 150 mg daily, laboratory studies revealed the following: total testosterone 11 ng/dL (goal <100 ng/dL) and estradiol 92 pg/mL (goal <200 pg/mL). Cholesterol panel and potassium level were normal.

**FIG. 3.**
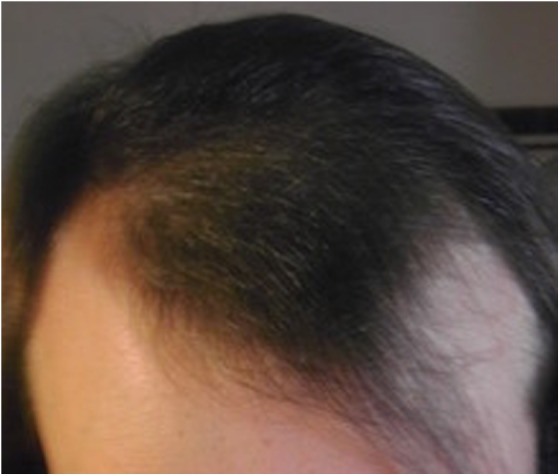
Scalp after ∼6 months of daily treatment with oral estradiol 5 mg and spironolactone 150 mg, frontal view. Serum testosterone level was 11 ng/dL.

**FIG. 4.**
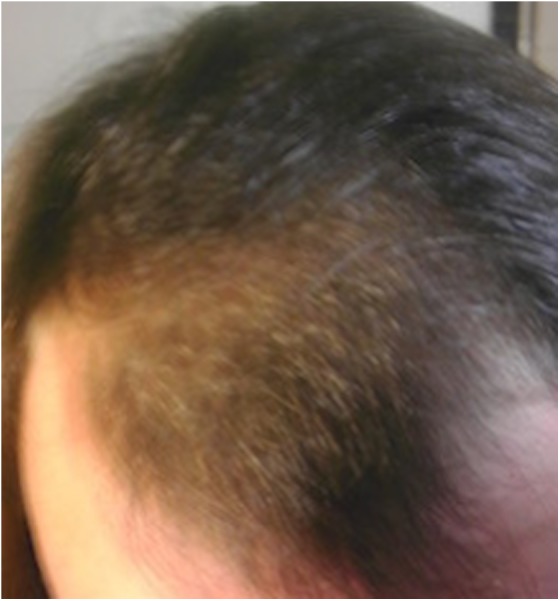
Scalp after ∼6 months of hormone treatment, top view.

After 6 months of treatment, the patient reported that she was happy with her regimen; she reported good effect in her physical appearance. In particular, she noted scalp hair regrowth as well as thickening of her existing hair (Figs. 3 and 4).

## Discussion

One goal of hormone therapy for male-to-female individuals is to reduce male-pattern hair growth.^6^ Based on our clinical experience, we have long suspected that there was reversal of male pattern baldness in transgender women on treatment to a degree dependent on age and likely other factors. The case presented demonstrates that hormone therapy for male-to-female transgender patients with estrogen and spironolactone can not only reduce male-pattern hair distribution but can also reverse previous effects of androgen on scalp hair patterns.

Spironolactone is an antiandrogen that acts primarily through competitive inhibition of androgen receptors; however, it has additional antiandrogen properties through inhibition of 5α-reductase activity, DHT binding, and androgen synthesis in the testes.^7,8^ In one small study examining the effect of spironolactone on genital skin 5α-reductase activity *in vivo* of hirsute women, spironolactone was found to have a direct inhibitory effect of 5α-reductase, thereby decreasing conversion of testosterone to DHT.^9^ Spironolactone is not commonly used to treat male pattern baldness, however, given the side effects of gynecomastia, impotence, and feminization of hair patterns.^10^ The antiandrogen effects of spironolactone that make it an undesirable option for treating male pattern baldness in men in fact make it a useful tool for achieving desired feminizing characteristics for transgender women, as seen in this case.

Estradiol plays a role in androgen inhibition. In males, estradiol has been shown to be one of the sex steroids (in addition to testosterone and DHT, however, through somewhat different mechanisms) capable of negative feedback on the hypothalamic-pituitary axis to inhibit luteinizing hormone secretion^11^ and thereby decrease testicular leydig cell production of testosterone.

We believe that the mechanism responsible for achieving scalp hair regrowth in transgender women is the suppression of testosterone to normal female levels. In our experience, this usually requires therapy with both spironolactone and estradiol. However, if transgender women treated with estrogen alone can achieve testosterone at normal female levels, we would expect to see scalp hair regrowth in these patients as well. Furthermore, it would be interesting to determine whether more scalp hair regrowth occurs over time as this patient continues hormone therapy.

## Abbreviation Used

DHT: dihydrotestosterone
